# Regional citrate anticoagulation in cardiac surgery patients at high risk of bleeding: a continuous veno-venous hemofiltration protocol with a low concentration citrate solution

**DOI:** 10.1186/cc11403

**Published:** 2012-06-27

**Authors:** Santo Morabito, Valentina Pistolesi, Luigi Tritapepe, Laura Zeppilli, Francesca Polistena, Emanuela Strampelli, Alessandro Pierucci

**Affiliations:** 1 Department of Nephrology and Urology, Hemodialysis Unit, Umberto I, Policlinico di Roma, "Sapienza" University, Viale del Policlinico, 155, 00161, Rome, Italy; 2 Department of Anesthesiology and Intensive Care, Cardiac Surgery ICU, Umberto I, Policlinico di Roma, "Sapienza" University, Viale del Policlinico, 155, 00161, Rome, Italy

## Abstract

**Introduction:**

Regional citrate anticoagulation (RCA) is a valid option in patients at high risk of bleeding who are undergoing continuous renal replacement therapy (CRRT). The aim of this study was to evaluate, in critically ill patients with severe acute kidney injury following cardiac surgery, the efficacy and safety of RCA-continuous veno-venous hemofiltration (CVVH) using a low concentration citrate solution.

**Methods:**

In high bleeding-risk cardiac surgery patients, we adopted, as an alternative to heparin or no anticoagulation, RCA-CVVH using a 12 mmol/l citrate solution. For RCA-CVVH settings, we developed a mathematical model to roughly estimate citrate load and calcium loss. In order to minimize calcium chloride supplementation, a calcium-containing solution was used as post-dilution replacement fluid.

Statistical analysis was performed using the Student t-test or analysis of variance (ANOVA) with post-hoc tests, Wilcoxon or Kruskal-Wallis tests for non-parametric analysis, and Kaplan-Meier survival analysis with Log Rank test.

**Results:**

Thirty-three patients (age 70.8 ± 9.5, Sequential Organ Failure Assessment (SOFA) score 13.9 ± 2.5) were switched to RCA-CVVH from no anticoagulation CRRT. Among them, 16 patients had been previously switched from heparin to no anticoagulation because of bleeding or heparin-related complications. RCA-CVVH filter life (49.8 ± 35.4 hours, median 41, 152 circuits) was significantly longer (*P *< 0.0001) when compared with heparin (30.6 ± 24.3 hours, median 22, 73 circuits) or no anticoagulation (25.7 ± 21.2 hours, median 20, 77 circuits). Target circuit and systemic Ca^++ ^were easily maintained (0.37 ± 0.09 and 1.18 ± 0.13 mmol/l), while the persistence of a mild metabolic acidosis required bicarbonate supplementation (5.8 ± 5.9 mmol/hours) in 27 patients. The probability of circuit running at 24, 48, 72 hours was higher during RCA-CVVH (*P *< 0.0001), with a lower discrepancy between delivered and prescribed CRRT dose (*P *< 0.0001). RCA was associated with a lower transfusion rate (*P *< 0.02). Platelet count (*P *= 0.012) and antithrombin III activity (*P *= 0.004) increased throughout RCA-CVVH, reducing the need for supplementation.

**Conclusions:**

RCA safely prolonged filter life while decreasing CRRT downtime, transfusion rates and supplementation needs for antithrombin III and platelets. In cardiac surgery patients with severe multiple organ dysfunction syndrome, the adoption of a 12 mmol/l citrate solution may provide a suboptimal buffers supply, easily overwhelmed by bicarbonate supplementation.

## Introduction

Continuous renal replacement therapies (CRRT) are widely adopted in the management of severe acute kidney injury (AKI) in critically ill patients with hemodynamic instability and multiple organ dysfunction syndrome (MODS) [1-3]. A potential drawback of CRRT is the need for prolonged anticoagulation to prevent clotting of the extracorporeal circuit [4]. Heparin is the standard choice but the incidence of bleeding is reported in up to 30% of renal replacement therapies [5-8] and it is well known that bleeding complications are associated with an increased risk of mortality in AKI patients [9]. Bleeding risk and/or the development of heparin-induced thrombocytopenia contributed to an increasing interest in alternative strategies [10-13]. Among them, regional citrate anticoagulation (RCA) seems to be a valid option in patients with a high bleeding risk [12,13]. Citrate provides anticoagulation by a process referred to as chelation of ionized calcium [12]. The depletion of ionized calcium interrupts clotting cascade activation at several stages [14]. Since citrate is a small molecule (MW 258 Da), the calcium-citrate complex is easily removed by diffusion and/or convection and systemic calcium infusion is thus required to replace the calcium lost in the effluent [15]. The citrate metabolic load derives from the balance between the prescribed citrate dose and the amount of citrate removed by filtration and/or dialysis [15]. Citrate returning to the patient is rapidly metabolized in bicarbonate mainly by the liver, but also by skeletal muscle and the renal cortex [12]. Reported issues with RCA include metabolic alkalosis and acidosis, hyper- and hyponatremia and hypocalcemia, but these complications are uncommon with an accurate monitoring of the procedure [12,16]. Known RCA protocols are characterized by variability in CRRT modality, citrate metabolic load and composition of citrate and CRRT solutions, in many cases customized and hospital pharmacy-formulated [17]. However, the availability of dedicated commercial solutions could help simplify protocols allowing to improve safety and to expand the use of RCA.

The aim of this study was to evaluate prospectively the efficacy and safety of a simplified RCA protocol adopting a low concentration citrate solution in critically ill patients with a high bleeding risk undergoing CRRT for AKI following cardiac surgery.

## Materials and methods

Starting in May 2008 we adopted RCA as an alternative to heparin or no anticoagulation (no-AC) in patients with a high risk of bleeding who were undergoing CRRT due to AKI following cardiac surgery. The present observational study was in agreement with the Declaration of Helsinki and written informed consent was obtained from either the patient or a close relative. Ethics Committee approval was not required for this observational study because all data reported, as well as anticoagulation method assignment, were part of our routine medical procedures and guidelines.

CRRT was performed using the Prismaflex system (Gambro Lundia AB, Lund, Sweden). Acrylonitrile sodium-metallyl-sulfonate (AN69ST) or polyarylethersulfone (PAES) hemofilter (Prismaflex ST100, 1 m^2^, or HF 1000, 1.15 m^2^, Gambro, Meyzieu, France) were used. A conventional heparin protocol (starting infusion 5 IU/kg/hour, adjusted to meet the target activated partial thromboplastin time (aPTT) ratio of approximately 1.5) was applied only to patients without active bleeding. Patients who fulfilled or developed any of the following criteria, defining a high bleeding risk, were assigned or switched to a no-AC regimen: platelet count < 50000/μl or heparin-induced thrombocytopenia, spontaneous or heparin associated bleeding, basal aPTT > 45 seconds, and surgery in the last 48 hours [18]. Filter clotting within 24 hours running time was considered as a criterion to switch from no-AC to RCA.

**Figure 1:**
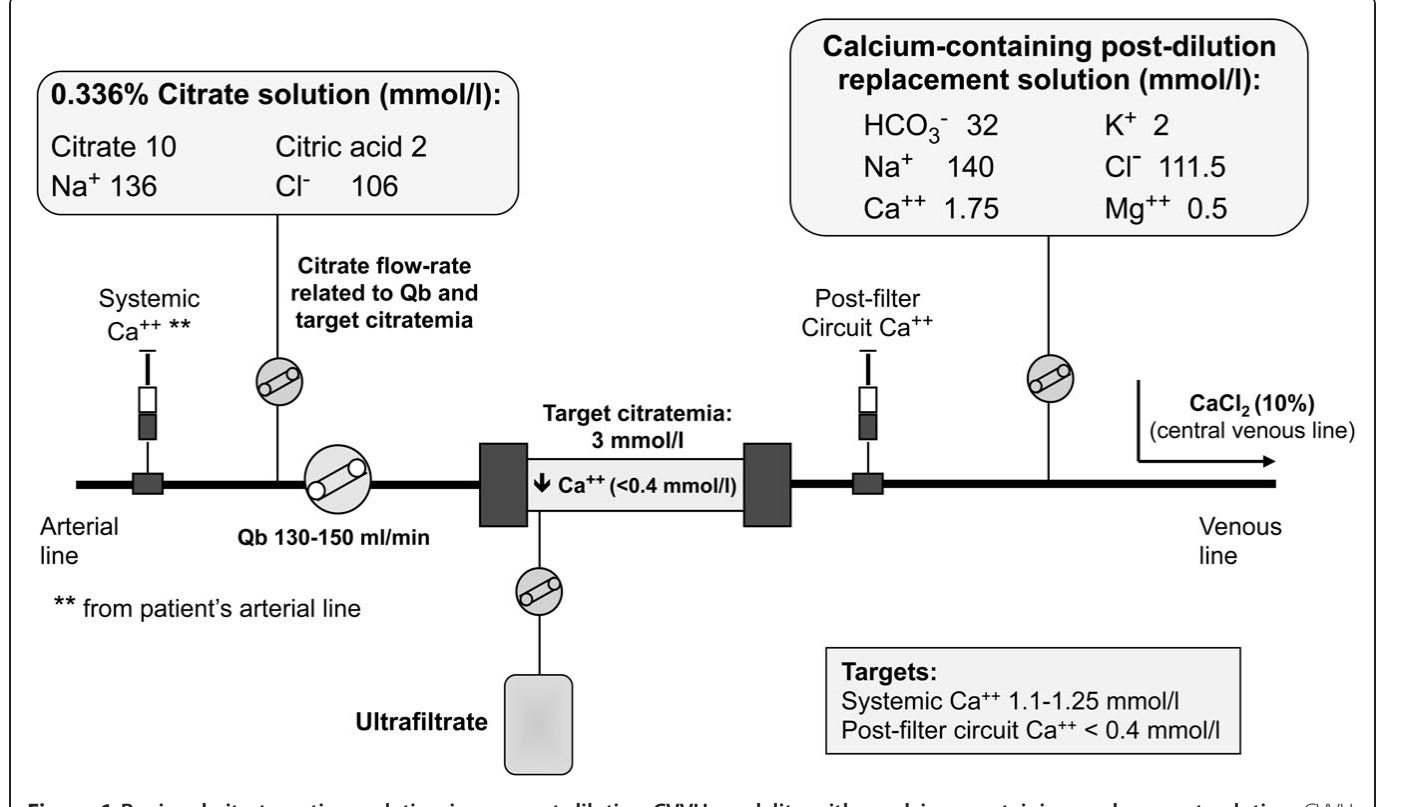
**Regional citrate anticoagulation in pre-post dilution CVVH modality with a calcium-containing replacement solution**. CVVH, continuous veno-venous hemofiltration.

Heparin and no-AC CRRT were performed in pre-dilution continuous veno-venous hemodiafiltration (CVVHDF) using a bicarbonate solution as dialysate and replacement fluid (HCO_3_^- ^32, Ca^++ ^1.75, Mg^++ ^0.5, K^+ ^2, Na^+ ^140, Cl^- ^111.5 mmol/l; Prismasol 2, Gambro, Sondalo, Italy) with a suggested dialysis dose corrected for pre-dilution (correction factor = blood flow rate/(blood flow rate + pre-dilution infusion rate)) of at least 25 ml/kg/hour. RCA was performed in CVVH modality (RCA-CVVH) using a pre-dilution citrate solution (trisodium citrate 10, citric acid 2, Na^+ ^136 mmol/l, Cl^- ^106 mmol/l; Prismocitrate 10/2, Gambro, Sondalo, Italy) and a post-dilution bicarbonate solution (Prismasol 2) (Figure 1).

In relation to blood flow rate (Qb), citrate solution rate was set to meet a circuit citrate concentration of 3 mmol/l and modified, if needed, to obtain circuit ionized calcium (c-Ca^++^) < 0.40 mmol/l (post-filter sample). Post-dilution bicarbonate solution rate was adjusted to achieve a total dialysis dose of 30 ml/kg/hour with the aim of ensuring a prescribed dialysis dose, corrected for pre-dilution, of at least 25 ml/kg/hour. Calcium chloride (CaCl_2 _10%) was infused in a separate central venous line to maintain systemic ionized calcium (s-Ca^++^) in the normal range (1.1 to 1.25 mmol/l). During RCA-CVVH, magnesium sulphate was infused as needed to avoid hypomagnesemia. To facilitate the initial RCA-CVVH settings, we used a mathematical model to roughly estimate metabolic citrate load, buffers balance (citrate and bicarbonate) and effluent calcium loss. The model, developed with the FileMaker database (FileMaker Inc, Santa Clara, CA, USA) and compatible with many portable devices, allowed easy parameter calculation at the patient's bedside. Assumed sieving coefficients (SC) were 0.9 for citrate, 1.0 for ionized calcium and bicarbonate. The input fields were as follows: Qb (ml/minute), citrate solution concentration (mmol/l), citrate solution flow rate (l/hour), bicarbonate and ionized calcium replacement solution concentration (mmol/l), post-dilution flow rate (l/hour), patient's bicarbonate and ionized calcium (mmol/l), patient's hematocrit (%) and serum protein (g/dl), net ultrafiltration rate (l/hour). Calculated output fields (corrected for pre-dilution when appropriate) were as follows: pre-filter estimated citrate blood concentration (mmol/l) calculated in plasma water ((citrate solution concentration x citrate flow rate)/(citrate flow rate + plasma water flow rate)), total effluent rate (l/hour), filtration fraction (%), estimated citrate metabolic load (mmol/hour) ((citrate solution concentration x citrate flow rate) - (effluent rate x estimated citrate blood concentration x SC)), CVVH buffers and calcium balance (mmol), and suggested CaCl_2 _infusion rate (ml/hour).

Serum electrolytes, including total Ca, P, K, Mg, coagulation parameters and complete blood count were assessed daily. Acid-base parameters and electrolytes (K^+^, Ca^++^) were measured by an arterial blood gases analyzer (GEM Premiere 4000, Instrumentation Laboratory UK Ltd, Warrington, UK) at least every four hours. Potassium and phosphate loss with CRRT was replaced with potassium chloride and sodium phosphate infusion. Total calcium/s-Ca^++ ^ratio (Calcium Ratio) > 2.5 was considered an indirect sign of citrate accumulation [19].

Reasons for stopping CRRT have been accurately reported after evaluation of monitor events and pressure alarms, recorded on the Prismaflex memory card. CRRT interruption due to coagulation was defined as an overt sign of circuit clotting or as a 100% increase of filter drop pressure (the difference between pre-filter and post-filter hydrostatic pressure). CRRT interruption for clinical reasons (that is, for evaluation of renal function recovery, modification of CRRT schedule during the recovery phase of AKI, patient mobilization, and so on), unrelated to circuit clotting, was classified and reported as scheduled CRRT stopping. Regardless of the anticoagulation modality, blood units transfused per day were recorded throughout the CRRT period.

### Statistical analysis

Data are reported as mean ± standard deviation (m ± SD). Statistical analysis for continuous variables was made using the Student t-test or analysis of variance (ANOVA) with Bonferroni post-hoc test. Non-parametric tests were performed using the Wilcoxon signed-rank test for related samples or Kruskal-Wallis test for independent samples. Circuit lifetime was evaluated with Kaplan-Meier survival analysis and survival curves distribution was compared with the Log Rank (Mantel-Cox) test. All tests were two-sided (significance level 5%). IBM SPSS statistical (19.0, SPSS Inc., USA) was used for all analyses.

## Results

Thirty-three high-bleeding-risk patients underwent RCA-CVVH due to AKI following cardiac surgery. In all cases RCA-CVVH was started because of early circuit clotting (< 24 hours) with no-AC CRRT. Among them, 16 patients had been previously switched from heparin CRRT to no-AC because of bleeding or heparin-related complications. Clinical characteristics of the patients at the time of starting CRRT and RCA-CVVH initial parameters are listed in Table 1. During RCA-CVVH, c-Ca^++ ^post-filter values, almost constantly < 0.40 mmol/l (0.37 ± 0.09, median 0.37), confirmed the adequacy of citrate flow rate in most cases (149/152 sessions) while an increase of the initial citrate infusion rate was required, for c-Ca^++ ^recurrently > 0.4 mmol/l, in only 3/152 sessions. Systemic Ca^++ ^was easily maintained in the normal range with few modifications of CaCl_2 _flow rate (1 to 2 within 24 hours). CaCl_2 _was infused at a mean rate of 2.38 ± 0.77 mmol/hour, which is equivalent to an amount of calcium element of 2.3 ± 0.7 g/day. Mean s-Ca^++ ^was 1.18 ± 0.13 mmol/l (median 1.18). No episodes of clinically relevant hypocalcemia or hypercalcemia were observed. Mean Calcium Ratio was 1.98 ± 0.2 (median 1.96, range 1.48 to 3.08). In one patient with cardiogenic shock, RCA-CVVH was stopped due to an indirect sign of citrate accumulation (Calcium Ratio = 3.08).

**Table 1:** Clinical characteristics of the patients at the time of starting CRRT and RCA-CVVH initial parameters.

Number = 33 (24 men, 9 women)	
Age (years)	70.8 ± 9.5 (range 46 to 85)
Creatinine (mg/dl)	2.5 ± 0.9
Blood urea nitrogen (mg/dl)	54.3 ± 26.2
Mean arterial pressure (mmHg)	72.5 ± 10.2
Oliguric AKI^a^	94%
Mechanical ventilation	100%
Total parenteral or enteral nutrition	100%
Use of vasopressors or inotropes	75.8%
APACHE II score	32.1 ± 4.6
SOFA score	13.9 ± 2.5
MELD score	18.7 ± 4.7
Bilirubin (mg/dl)	1.68 ± 1.98
Cardiovascular surgery:	
Coronary artery bypass grafting	33.3%
Coronary artery bypass grafting + valvular surgery	27.3%
Ascending aorta replacement	24.2%
Valvular surgery	15.2%

**RCA-CVVH initial parameters**^b^	

Prescribed dialysis dose, corrected for pre-dilution (ml/kg/hour)	28.1 ± 2.9
Blood flow rate (ml/minute)	135.7 ± 14.6
Pre-dilution citrate solution flow rate (l/hour)	1.69 ± 0.23
Post-dilution bicarbonate solution flow rate (l/hour)	0.77 ± 0.17
Calcium chloride 10% (mmol/hour)	2.38 ± 0.77
Citrate infusion rate (mmol/hour)	20.3 ± 2.8
Estimated citrate load (mmol/hour)	11.5 ± 2

Data are expressed as mean ± SD or percentage. ^a^According to AKIN criteria (Crit Care 2007; 11:R31). ^b^Data derived from the first RCA-CVVH session for each patient. AKI, acute kidney injury; AKIN, Acute Kidney Injury Network; APACHE, Acute Physiology and Chronic Health Evaluation; CRRT, continuous renal replacement therapy; MELD, Model for End-Stage Liver Disease; RCA-CVVH, regional citrate anticoagulation-continuous veno-venous hemofiltration; SD, standard deviation; SOFA, Sequential Organ Failure Assessment.

**Figure 2:**
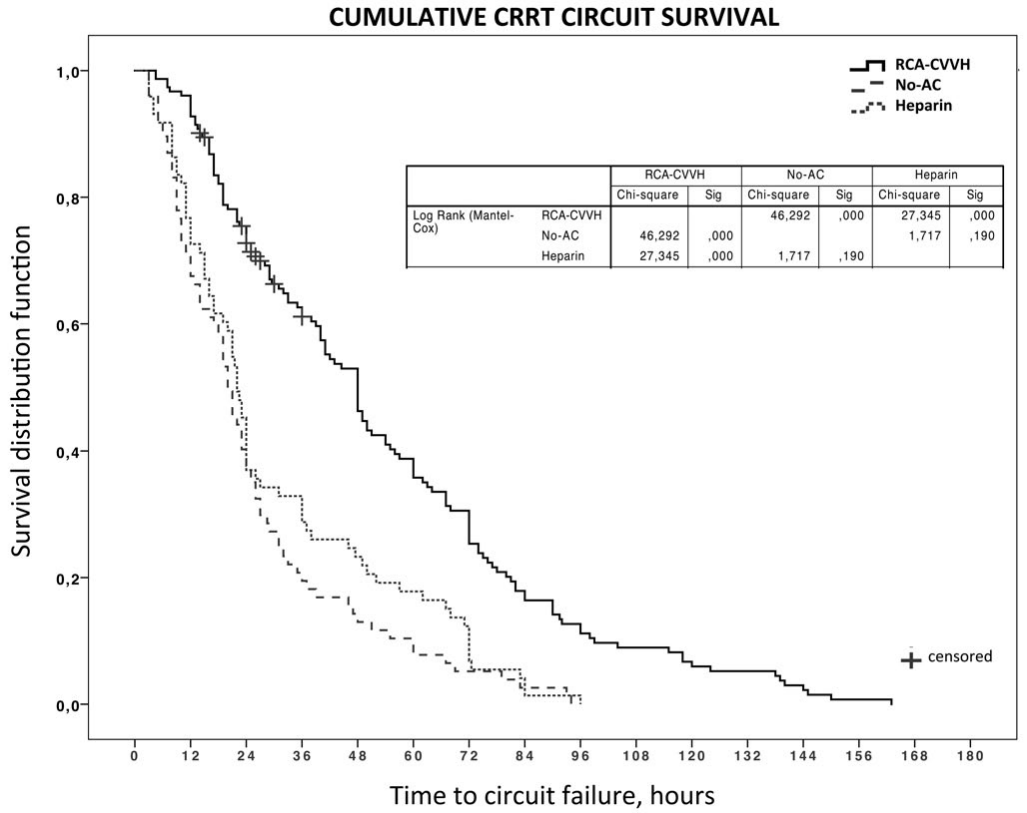
**Kaplan-Meier curves of circuit lifetime probability, according to different anticoagulation modalities, derived from analysis of scheduled and unscheduled CRRT stopping for any cause**. Scheduled CRRT stopping has been censored. Survival curves distribution has been compared with Log Rank (Mantel-Cox) test (*P *< 0.0001). CRRT, continuous renal replacement therapy.

One hundred fifty-two circuits were used in RCA-CVVH with a filter life of 49.8 ± 35.4 hours (median 41, range 4.5 to 163, total 7,570) (Table 2). Excluding scheduled CRRT stopping, circuit lifetime was 52.2 ± 36.1 hours (median 48). Before starting RCA, we used 73 heparin circuits in 16 patients and 77 no-AC circuits in 33 patients with a filter life of 30.6 ± 24.3 hours (median 22, range 3 to 96, total 2,233) and 25.7 ± 21.2 hours (median 20, range 3 to 94, total 1,980), respectively (Table 2), in both cases significantly shorter than RCA-CVVH (*P *= 0.0001). CRRT stopping causes and circuits running at 24, 48, 72 hours are reported in Table 2. RCA-CVVH did not stop in any case for filter clotting and filter drop pressure showed slight increments over the course of RCA-CVVH sessions (Δ filter drop pressure 11.8% ± 4.7% after 48 hours running time, median 7.4%). Only one circuit was replaced after 91 hours due to the presence of clots in the venous drip chamber. For each anticoagulation modality, Kaplan-Meier curves of circuit lifetime probability, derived from analysis of scheduled and unscheduled CRRT interruptions due to any cause, are displayed in Figure 2. The discrepancy between delivered versus prescribed CRRT dose, calculated as Δ-dose, was -4.7% during RCA-CVVH, significantly lower than heparin and no-AC modalities (-13% and -12.7% respectively, *P *< 0.0001). Prescribed dialysis dose, corrected for pre-dilution, was comparable among different anticoagulation modalities (Table 2). Delivered dialysis dose during RCA-CVVH (25.6 ± 4.9 ml/kg/hour) was significantly higher than that achieved with heparin (23.7 ± 7.2 ml/kg/hour, *P *= 0.016) and no-AC (23.1 ± 8 ml/kg/hour, *P *< 0.001) modalities (Table 2). During RCA-CVVH circuits consumption was 0.48/day, significantly lower if compared with heparin (0.78/day) or no-AC (0.93/day) (*P *< 0.0001). The main metabolic and electrolyte parameters for the first four days and for the last day of RCA-CVVH are shown in Table 3.

**Table 2:** Circuit lifetime, CRRT stopping causes and prescribed versus delivered dialysis dose according to different anticoagulation modalities.

	RCA (*n *= 152)	Heparin (*n *= 73)	No AC (*n *= 77)
**CIRCUIT LIFETIME**			

Mean ± SD (hours)	49.8 ± 35.4***	30.6 ± 24.3	25.7 ± 21.2
Median (hours)	41	22	20
> 24 hours	74%	45%	40%
> 48 hours	41%	25%	14%
> 72 hours	27%	12%	5%

**CRRT STOPPING CAUSES**			

CVC malfunction	34.9%	17.8%	15.6%
Alarm handling/technical issues	23.7%	12.3%	2.6%
Scheduled	19.7%	0%	1.3%
Medical procedures	13.8%	2.8%	3.9%
Clotting	0%	61.6%	68.8%
Unidentified	7.9%	5.5%	7.8%

**DIALYSIS DOSE**^a^			

Prescribed dose (ml/kg/hour)	26.8 ± 3.8	27.3 ± 4.7	26.6 ± 7.1
Delivered dose (ml/kg/hour)	25.6 ± 4.9**	23.7 ± 7.2	23.1 ± 8
Delta dose (%)	4.7 ± 12.1***	13 ± 20.5	12.7 ± 19.1

Data are expressed as mean ± SD or percentage. ^a^Corrected for predilution. Statistical comparison among different anticoagulation modalities: ANOVA with Bonferroni post-hoc test. ***P < 0.0001; **P < 0.02. AC, anticoagulation; ANOVA, analysis of variance; CRRT, continuous renal replacement therapy; CVC, central venous catheter; n, number; RCA, regional citrate anticoagulation; SD, standard deviation.

**Table 3:** Main metabolic and electrolyte parameters throughout RCA-CVVH days.

	Days on RCA				
	1	2	3	4	Last day
Systemic Ca^++ ^(mmol/l)	1.2 (1.09-1.36)	1.2 (1.14-1.25)	1.19 (1.15-1.24)	1.16 (1.12-1.26)	1.19 (1.13-1.24)
Circuit Ca^++ ^(mmol/l)	0.39 (0.33-0.43)	0.37 (0.31-0.4)	0.32 (0.28-0.37)**	0.35 (0.31-0.39)	0.34 (0.32-0.39)
Systemic sodium (mmol/l)	136 (134-139.2)	135 (133-138)	134 (132-138)*	134 (131.7-136)*	135 (134-136)
Estimated citrate load (mmol/hour)	11.3 (10.1-12.4)	11.3 (10.2-12.3)	11.3 (10.1-12.5)	11.3 (10.2-12.5)	10.7 (10.1-11.9)
Calcium Ratio	1.88 (1.78-2.04)	1.96 (1.87-2.04)	1.96 (1.84-2.1)	1.92 (1.82-2.1)	2 (1.89-2.08)
pH (units)	7.4 (7.35-7.43)	7.4 (7.36-7.42)	7.4 (7.34-7.43)	7.4 (7.35-7.43)	7.41 (7.37-7.43)
Systemic bicarbonates (mmol/l)	22.9 (20.6-23.9)	22 (20.9-22.8)	22 (20.7-23.2)	21.4 (20.2-23.3)	22 (20.4-23.2)
Base excess	-3 (-4.7 to -1.1)	-3.2 (-3.8 to -2)	-3.1 (-4.1 to -2)	-3 (-3.5 to -1.6)	-2.5 (-4 to -1)
Systemic lactate (mmol/l)	1.3 (1-1.8)	1.2 (0.9-1.5)	1.05 (0.8-1.25)	1 (0.9-1.3)	1.1 (0.7-1.65)

Data are expressed as median (interquartile range). All points of considered parameters were not significant, except for time day 3 versus day 1 of circuit Ca^++ ^(** *P *< 0.02) and for time day 3 and 4 versus day 1 of systemic sodium (* *P *< 0.05). RCA-CVVH, regional citrate anticoagulation-continuous veno-venous hemofiltration.

In 27 out of 33 patients, the persistence of a mild metabolic acidosis during RCA-CVVH, unrelated to citrate accumulation, required additional NaHCO_3 _infusion (5.8 ± 5.9 mmol/hour). In all patients Mg^++ ^levels were corrected with magnesium sulphate continuous infusion (3 g/day).

**Figure 3:**
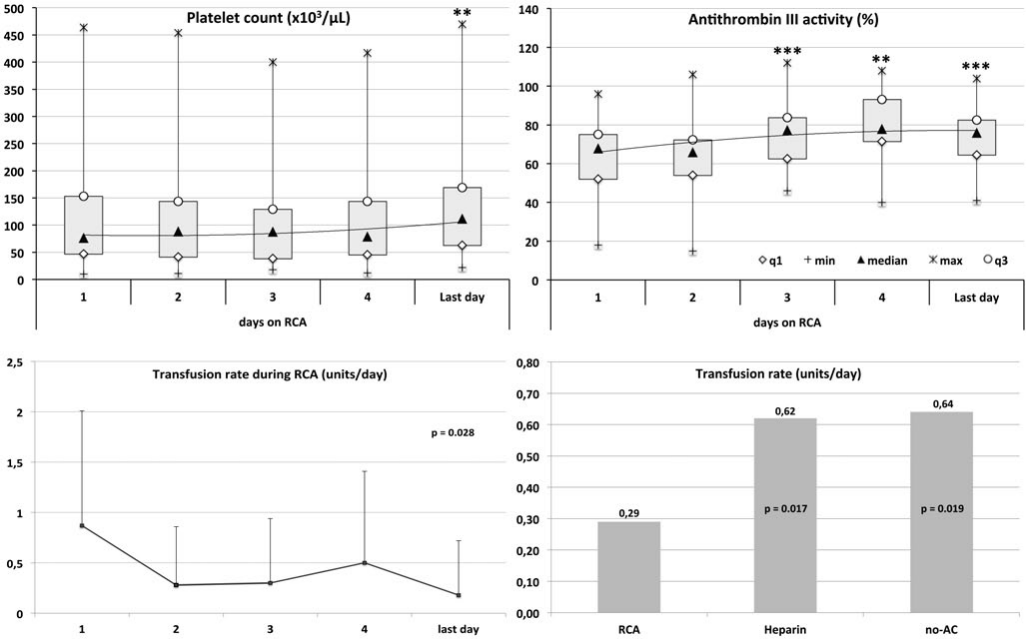
Platelet count and antithrombin III throughout days of RCA-CVVH (comparison versus day 1, ** *P *< 0.02; *** *P *< 0.005). Data are expressed as median, interquartile range (q1 to q3), minimum (min), maximum (max). On the bottom, transfusion rate (units/day) during RCA-CVVH days and comparison of transfusion rates among different anticoagulation modalities. RCA-CVVH, regional citrate anticoagulation-continuous veno-venous hemofiltration.

Platelet count and antithrombin III (AT-III) activity increased throughout RCA days (*P *= 0.012 and *P *= 0.004, respectively) allowing us to stop supplementation if previously required (Figure 3). During RCA-CVVH no patients had bleeding complications and the transfusion rate was lower compared with heparin (0.29 versus 0.62, *P *= 0.017) or no-AC (0.29 versus 0.64 blood units/day, *P *= 0.019) (Figure 3). Thirty-day survival was 66.7% while survival at discharge from the hospital was 45.5%. At the time of discharge, renal function recovery, allowing the cessation of RRT, was observed in 13 out of 15 survivors (86.7%).

## Discussion

Among the key problems of CRRT, the need for prolonged anticoagulation is its most important drawback [4]. The incidence of bleeding complications during RRT is extremely variable among different patient populations but, in any case, the incidence of major bleeding is not infrequent and cannot be neglected. Therefore, alternative methods of anticoagulation should be more widely adopted and, among potential alternatives to systemic anticoagulation, RCA is the most promising. Indeed, several studies reported better filter survival and/or fewer bleeding events with RCA, compared to unfractioned heparin [20-23]. A recent meta-analysis, including six randomized studies, confirmed that RCA was able to prolong circuit life and to reduce the risk of bleeding with a pooled risk ratio of 0.28 in comparison to the control group [24]. The aim of the present study was to evaluate the efficacy and safety of a simplified protocol of RCA-CVVH in patients undergoing CRRT due to AKI following cardiac surgery. In this selected population, the anticoagulation strategy was intended to reduce bleeding complications maintaining, at the same time, an adequate circuit lifespan to minimize downtime periods. All patients received RCA-CVVH because of a high risk of bleeding, contraindicating heparin, or because of heparin-related complications (bleeding, thrombocytopenia). In any case, RCA-CVVH was started after an attempt to perform CRRT without anticoagulation. In this regard, in the Acute Renal Failure Trial Network study more than 50% of CVVHDF treatments were performed without anticoagulation [25]. However, in the same study the prescribed dialysis dose was, in the best case, delivered in only 70% of the patients and it is well known that filter clotting, together with vascular access malfunction, is the main cause of discrepancy between the prescribed and delivered dose in CRRT [25]. The assessment of any difference in filter lifespan and CRRT downtime was a secondary endpoint of our observational study, with consequent limitations, mainly related to the comparison of anticoagulation modalities at different stages in the course of critical illness and to the recruitment to switch to RCA-CVVH after failure of no-AC. In any case, taking account of these limitations, the switch from no-AC to RCA-CVVH allowed us to prolong filter life significantly and to minimize treatment downtime. Indeed, the delivered dose in RCA-CVVH was about 95% of the prescribed dose, with a dose discrepancy significantly lower than that observed during heparin or no-AC CRRT. Furthermore, RCA-CVVH was associated with a significantly lower need for filter set replacement. Although cost analysis was not performed in our study, it has been reported that a longer filter life during RCA could play a role in minimizing the total CRRT cost compared to heparin anticoagulation [26,27]. On the other hand, indirect costs, such as platelet and red cell transfusions, as well as AT-III supplementation, can also be taken into account for cost analysis. In the present study, platelet count and AT-III activity increased throughout the days of RCA allowing us to stop supplementation where it was formerly required. Moreover, as already reported by other authors [20-23], no patients had bleeding complications during RCA-CVVH and the transfusion rate was significantly lower if compared with the other anticoagulation modalities. Regarding reasons for stopping CRRT, Kutsogiannis *et al. *reported, in 30 patients randomly assigned to heparin or citrate, a less frequent occurrence of circuit clotting during RCA (16.7% versus 53.5%, *P *= 0.002) [21]. In our study, RCA-CVVH did not stop in any case because of filter clotting, as confirmed by the stability of filter drop pressure recorded throughout the circuit running time.

Regarding a potential impact upon survival, most randomized studies comparing RCA with heparin anticoagulation are too small to demonstrate a difference in clinical outcome [13]. However, a recent study including 200 critically ill patients receiving CRRT, randomly assigned to low molecular weight heparin (LMWH) (nadroparin) or RCA, showed an unexpected 15% absolute increase in three-month survival, seemingly not justified by a lower incidence of bleeding [28]. In the same study, post-hoc analysis showed that RCA may be particularly beneficial in specific clinical conditions (surgery, sepsis, severe MODS, younger age). To explain these findings, it has been hypothesized that local hypocalcemia during RCA might reduce the release of inflammatory mediators from cells adhered to the hemofilter membrane [28]. Although the present study was not aimed at comparing survival among different anticoagulation strategies, in our selected population of cardiac surgery patients, receiving RCA-CVVH for AKI and severe MODS, survival at the time of discharge was about 45%, comparable to that reported by Oudemans-van Straaten in the citrate group [28].

Despite several reports about the efficacy and safety of RCA, the diffusion of this method of anticoagulation appears relatively limited, ranging from about 10% (B.E.S.T. kidney survey) [1] to 20% (Acute Renal Failure Trial Network study) [25] of CRRT treatments. Probably, among different reasons, RCA has not yet gained widespread application because of the complexity of early protocols and because of concerns about metabolic or electrolyte complications. With the aim of simplifying RCA handling, we adopted a protocol of pre-post dilution CVVH in which citrate was used as the anticoagulation solution as well as pre-dilution replacement fluid. The adoption of the CVVH modality allowed us to use only two different solutions (citrate + conventional replacement fluid) and to avoid zero calcium dialysate. Furthermore, to reach the prescribed dialysis dose and to minimize the amount of calcium supplementation, we introduced the novelty of adopting a calcium-containing post-dilution replacement fluid (1.75 mmol/l). Indeed, the amount of CaCl_2 _infused in a separate line was lower than that reported elsewhere [20,29], and the use of a calcium-containing post-dilution replacement fluid was not associated with drip chamber clotting in the venous line. Moreover, the use of a mathematical model to roughly estimate citratemia and calcium balance allowed us to easily calculate the initial setting of the RCA-CVVH parameters minimizing nurse workload related to the need for additional interventions. In particular, estimation of the initial setting of the CaCl_2 _infusion required only one or two adjustments in the first 24 hours of each session and resulted in the avoidance of complications related to hypo- or hypercalcemia. Mathematical models, developed to calculate the volume of citrate infusion required to achieve the target Ca^++ ^in the extracorporeal circuit and to restore the total calcium level have been recently proposed and validated [30,31]. Regarding metabolic or electrolyte complications, old RCA protocols were characterized by extreme variability in the citrate solution composition, in most cases requiring the adoption of customized low sodium concentration dialysate or replacement fluid [17,32]. However, alkalosis and hypernatremia, although representing potential RCA complications, are rarely observed with the appropriate combination of citrate and dialysate (and/or replacement fluid) solutions. In particular, alkalosis may be observed only in the case of imbalance between the supply of buffers (citrate and bicarbonate) and citrate/bicarbonate removal by ultrafiltration and/or dialysis (inappropriate combination of solutions and/or inadequate RCA-CRRT parameters setting). In this regard, strategies for the prevention of citrate accumulation should be targeted to decrease citrate administration, through the use of a low blood flow rate, and to increase citrate clearance, through optimization of convective and/or diffusive dialysis dose. Mariano *et al. *showed that a careful RCA strategy, targeted to reduce citrate load, ensured metabolic tolerance also in severe septic shock patients [33]. The protocol adopted in the present study provided an adequate RCA without electrolyte derangements. Furthermore, the citrate load was below the lowest range reported until now by other authors (33) and allowed us to prevent citrate accumulation in all but one patient. However, on the other side, the low amount of citrate delivered to the patient was associated, in most cases, with a suboptimal buffers supply. As a consequence, despite optimization of CVVH parameters (that is, citrate infusion rate and/or post-dilution bicarbonate flow rate) the persistence of a mild metabolic acidosis required additional bicarbonate infusion. Comparable findings, regarding the need for additional bicarbonate, have been reported by Hetzel performing CVVH with a 13 mmol/l citrate solution [23]. Therefore, adopting a very low concentration citrate solution requires further refinements to optimize the buffers balance. Our purpose was to evaluate the use of a more concentrated bicarbonate replacement fluid in order to customize the buffers supply in individual patients, through the modulation of the post-dilution flow rate according to their acid-base status. On the other hand, the use of a higher citrate concentration (18 mmol/l), reported by Tolwani [34], provided an appropriate acid-base balance but required, to avoid alkalosis, a lower than usual dialysate bicarbonate concentration (25 mmol/l). Morgera *et al.*, performing CVVHDF with a high concentration citrate solution (136 mmol/l), combined with a low sodium and bicarbonate dialysate (133 and 20 mmol/l, respectively), were able to modulate acid-base status by modifying either the dialysate or the blood flow rate [35].

## Conclusions

The regional citrate anticoagulation protocol adopted in our study appeared safe, easy to apply and effective in preventing circuit clotting, thus minimizing CRRT downtime in critically ill cardiac surgery patients with AKI and MODS. Furthermore, RCA allowed us to ensure an adequate filter life and to decrease the transfusion rate, as well as the supplementation need for AT-III and platelets, without bleeding complications. In our opinion, RCA should be worthy of more consideration as the first choice anticoagulation modality in critically ill patients undergoing CRRT. However, in cardiac surgery patients with severe MODS, the adoption of a 12 mmol/l citrate solution may have the drawback of a suboptimal buffers supply, easily overwhelmed by bicarbonate supplementation. In forthcoming studies, our efforts will be focused on improving the RCA-CVVH protocol to further minimize the need for calcium supplementation and to better customize buffers balance in the individual patient, according to acid-base status and through the use of different combinations of citrate solutions and post-dilution replacement fluids.

## Key messages

• RCA-CVVH with a low concentration citrate solution ensured an adequate filter life and allowed a decrease in transfusion rates. Furthermore, platelet count and AT-III activity increased throughout the RCA-CVVH days.

• RCA was able to limit the discrepancy between prescribed and delivered dose by minimizing CRRT downtime.

• During RCA-CVVH, the need for calcium chloride supplementation may be reduced, in the absence of venous drip chamber clotting, by the use of a calcium-containing post-dilution replacement solution.

• In cardiac surgery patients undergoing RCA-CVVH for AKI associated with severe MODS, the use of a particularly low concentration citrate solution may be associated with the drawback of a 'suboptimal' buffers supply, easily overwhelmed by bicarbonate supplementation.

## Abbreviations

AC: anticoagulation; AKI: acute kidney injury; AKIN: Acute Kidney Injury Network; ANOVA: analysis of variance; AN69ST: acrylonitrile sodium-metallyl-sulfonate; APACHE II: Acute Physiology and Chronic Health Evaluation II; AT-III: antithrombin III; c-Ca^++^: circuit ionized calcium; CRRT: continuous renal replacement therapy; CVVH: continuous veno-venous hemofiltration; CVVHDF: continuous veno-venous hemodiafiltration; LMWH: low molecular weight heparin; MELD: Model for End-Stage Liver Disease; MODS: Multiple Organ Dysfunction Syndrome; MW: molecular weight; PAES: polyarylethersulfone; Qb: blood flow rate; RCA: regional citrate anticoagulation; RRT: renal replacement therapy; s-Ca^++^: systemic ionized calcium; SOFA: Sequential Organ Failure Assessment.

## Competing interests

The authors declare that they have no competing interests.

## Authors' contributions

SM and VP were involved in the conception, design, analysis and interpretation of data, drafting the article and revising it critically for important intellectual content and final approval of the version to be published. LT was involved in revising the manuscript for important intellectual content and final approval of the version to be published. LZ and FP provided substantial contributions to data collection and to preparing the manuscript. ES provided substantial contributions to data collection. AP contributed to the interpretation of data and critically revised the manuscript for important intellectual content and final approval of the version to be published. SM and VP contributed equally to the work and are both considered first authors. LT and AP contributed equally to the work and are both considered senior authors. All authors read and approved the final manuscript.

## References

[B1] Uchino S Bellomo R Morimatsu H Morgera S Schetz M Tan I Bouman C Macedo E Gibney N Tolwani A Oudemans-van Straaten H Ronco C Kellum JA Continuous renal replacement therapy: a worldwide practice survey. The beginning and ending supportive therapy for the kidney (B.E.S.T. kidney) investigators Intensive Care Med 2007 33 1563 1570 10.1007/s00134-007-0754-4 17594074

[B2] Morabito S Pistolesi V Cibelli L Pierucci A Continuous renal replacement therapies (CRRT) will remain the most widely adopted dialysis modality in the critically ill G Ital Nefrol 2009 26 13 21 19255959

[B3] Prowle JR Bellomo R Continuous renal replacement therapy: recent advances and future research Nat Rev Nephrol 2010 6 521 529 10.1038/nrneph.2010.100 20644583

[B4] Mehta RL Anticoagulation during continuous renal replacement therapies ASAIO J 1994 40 931 935 7858328

[B5] Ward DM Mehta RL Extracorporeal management of acute renal failure patients at high risk of bleeding Kidney Int 1993 43 Suppl 41 S237 S244 8320930

[B6] Brophy PD Somers MJ Baum MA Symons JM McAfee N Fortenberry JD Rogers K Barnett J Blowey D Baker C Bunchman TE Goldstein SL Multicentre evaluation of anti-coagulation in patients receiving continuous renal replacement therapy (CRRT) Nephrol Dial Transplant 2005 20 1416 1421 10.1093/ndt/gfh817 15855212

[B7] Oudemans-van Straaten HM Wester JP de Pont AC Schetz MR Anticoagulation strategies in continuous renal replacement therapy: can the choice be evidence based? Intensive Care Med 2006 32 188 202 10.1007/s00134-005-0044-y 16453140

[B8] Tolwani AJ Wille KM Anticoagulation for continuous renal replacement therapy Semin Dial 2009 22 141 145 10.1111/j.1525-139X.2008.00545.x 19426417

[B9] Fiaccadori E Maggiore U Clima B Melfa L Rotelli C Borghetti A Incidence, risk factors, and prognosis of gastrointestinal hemorrhage complicating acute renal failure Kidney Int 2001 59 1510 1519 10.1046/j.1523-1755.2001.0590041510.x 11260415

[B10] Tan HK Baldwin I Bellomo R Continuous veno-venous hemofiltration without anticoagulation in high-risk patients Intensive Care Med 2000 26 1652 1657 10.1007/s001340000691 11193272

[B11] Morabito S Guzzo I Solazzo A Muzi L Luciani R Pierucci A Continuous renal replacement therapies: anticoagulation in the critically ill at high risk of bleeding J Nephrol 2003 16 566 571 14696760

[B12] Davenport A Tolwani A Citrate anticoagulation for continuous renal replacement therapy (CRRT) in patients with acute kidney injury admitted to the intensive care unit NDT Plus 2009 2 439 447 10.1093/ndtplus/sfp136 PMC4421325 25949376

[B13] Oudemans-van Straaten HM Kellum JA Bellomo R Clinical review: anticoagulation for continuous renal replacement therapy - heparin or citrate? Crit Care 2011 15 202 21345279 10.1186/cc9358 PMC3222015

[B14] Abramson S Niles JL Anticoagulation in continuous renal replacement therapy Curr Opin Nephrol Hypertens 1999 8 701 707 10.1097/00041552-199911000-00009 10630816

[B15] Mariano F Morselli M Bergamo D Hollo Z Scella S Maio M Tetta C Dellavalle A Stella M Triolo G Blood and ultrafiltrate dosage of citrate as a useful and routine tool during continuous venovenous haemodiafiltration in septic shock patients Nephrol Dial Transplant 2011 26 3882 3888 10.1093/ndt/gfr106 21385861

[B16] Fall P Szerlip HM Continuous renal replacement therapy: cause and treatment of electrolyte complications Semin Dial 2010 23 581 585 10.1111/j.1525-139X.2010.00790.x 21166876

[B17] Tolwani AJ Campbell RC Schenk MB Allon M Warnock DG Simplified citrate anticoagulation for continuous renal replacement therapy Kidney Int 2001 60 370 374 10.1046/j.1523-1755.2001.00809.x 11422774

[B18] Morabito S Guzzo I Solazzo A Muzi L Pistolesi V Pierucci A Acute renal failure following cardiac surgery G Ital Nefrol 2006 23 Suppl 36 S52 S60 17068730

[B19] Hetzel GR Taskaya G Sucker C Hennersdorf M Grabensee B Schmitz M Citrate plasma levels in patients under regional anticoagulation in continuous venovenous hemofiltration Am J Kidney Dis 2006 48 806 811 10.1053/j.ajkd.2006.07.016 17060000

[B20] Monchi M Berghmans D Ledoux D Canivet JL Dubois B Damas P Citrate vs. heparin for anticoagulation in continuous venovenous hemofiltration: a prospective randomized study Intensive Care Med 2004 30 260 265 10.1007/s00134-003-2047-x 14600809

[B21] Kutsogiannis DJ Gibney RT Stollery D Gao J Regional citrate versus systemic heparin anticoagulation for continuous renal replacement in critically ill patients Kidney Int 2005 67 2361 2367 10.1111/j.1523-1755.2005.00342.x 15882280

[B22] Betjes MG van Oosterom D van Agteren M van de Wetering J Regional citrate versus heparin anticoagulation during venovenous hemofiltration in patients at low risk for bleeding: similar hemofilter survival but significantly less bleeding J Nephrol 2007 20 602 608 17918147

[B23] Hetzel GR Schmitz M Wissing H Ries W Schott G Heering PJ Isgro F Kribben A Himmele R Grabensee B Rump LC Regional citrate versus systemic heparin for anticoagulation in critically ill patients on continuous venovenous haemofiltration: a prospective randomized multicentre trial Nephrol Dial Transplant 2011 26 232 239 10.1093/ndt/gfq575 20876598

[B24] Zhang Z Hongying N Efficacy and safety of regional citrate anticoagulation in critically ill patients undergoing continuous renal replacement therapy Intensive Care Med 2012 38 20 28 10.1007/s00134-011-2438-3 22124775

[B25] VA/NIH Acute Renal Failure Trial Network Palevsky PM Zhang JH O'Connor TZ Chertow GM Crowley ST Choudhury D Finkel K Kellum JA Paganini E Schein RM Smith MW Swanson KM Thompson BT Vijayan A Watnick S Star RA Peduzzi P Intensity of renal support in critically ill patients with acute kidney injury N Engl J Med 2008 359 7 20 18492867 10.1056/NEJMoa0802639 PMC2574780

[B26] Morgera S Scholle C Voss G Haase M Vargas-Hein O Krausch D Melzer C Rosseau S Zuckermann-Becker H Neumayer HH Metabolic complications during regional citrate anticoagulation in continuous venovenous hemodialysis: single-center experience Nephron Clin Pract 2004 97 c131 c136 10.1159/000079171 15331942

[B27] Park JS Kim GH Kang CM Lee CH Regional anticoagulation with citrate is superior to systemic anticoagulation with heparin in critically ill patients undergoing continuous venovenous hemodiafiltration Korean J Intern Med 2011 26 68 75 10.3904/kjim.2011.26.1.68 21437165 PMC3056258

[B28] Oudemans-van Straaten HM Bosman RJ Koopmans M van der Voort PH Wester JP van der Spoel JI Dijksman LM Zandstra DF Citrate anticoagulation for continuous venovenous hemofiltration Crit Care Med 2009 37 545 552 10.1097/CCM.0b013e3181953c5e 19114912

[B29] Bihorac A Ross EA Continuous venovenous hemofiltration with citrate-based replacement fluid: efficacy, safety, and impact on nutrition Am J Kidney Dis 2005 46 908 918 10.1053/j.ajkd.2005.08.010 16253732

[B30] Brain M Parkes S Fowler P Robertson I Brown A Calcium flux in continuous venovenous haemodiafiltration with heparin and citrate anticoagulation Crit Care Resusc 2011 13 72 81 21627574

[B31] Brandl M Strobl K Hartmann J Kellner K Posnicek T Falkenhagen D A target-orientated algorithm for regional citrate-calcium anticoagulation in extracorporeal therapies Blood Purif 2011 33 7 20 22085810 10.1159/000332394

[B32] Mariano F Triolo G Anticoagulation of extracorporeal circuit in critically ill patients G Ital Nefrol 2007 24 34 42 17342691

[B33] Mariano F Tedeschi L Morselli M Stella M Triolo G Normal citratemia and metabolic tolerance of citrate anticoagulation for hemodiafiltration in severe septic shock burn patients Intensive Care Med 2010 36 1735 1743 10.1007/s00134-010-1909-2 20480135

[B34] Tolwani AJ Prendergast MB Speer RR Stofan BS Wille KM A practical citrate anticoagulation continuous venovenous hemodiafiltration protocol for metabolic control and high solute clearance Clin J Am Soc Nephrol 2006 1 79 87 17699194 10.2215/CJN.00040505

[B35] Morgera S Schneider M Slowinski T Vargas-Hein O Zuckermann-Becker H Peters H Kindgen-Milles D Neumayer HH A safe citrate anticoagulation protocol with variable treatment efficacy and excellent control of the acid-base status Crit Care Med 2009 37 2018 2024 10.1097/CCM.0b013e3181a00a92 19384210

